# Factors Influencing Participation in Clinical Trials: Emergency Medicine vs. Other Specialties

**DOI:** 10.5811/westjem.2017.5.33827

**Published:** 2017-07-17

**Authors:** Anita Kurt, Hope M. Kincaid, Charity Curtis, Lauren Semler, Matthew Meyers, Melanie Johnson, Beth A. Careyva, Brian Stello, Timothy J. Friel, Mark C. Knouse, John C. Smulian, Jeanne L. Jacoby

**Affiliations:** * Lehigh Valley Health Network, Department of Emergency Medicine Research, Allentown, Pennsylvania; † Lehigh Valley Health Network, Network Office of Research and Innovation, Allentown, Pennsylvania; ‡ Lehigh Valley Health Network, Department of Family Medicine, Allentown, Pennsylvania; § Lehigh Valley Health Network, Department of Medicine, Allentown, Pennsylvania; ¶ Lehigh Valley Health Network, Division of Infectious Diseases, Allentown, Pennsylvania; || Lehigh Valley Health Network, Division of Maternal Fetal Medicine, Allentown, Pennsylvania

## Abstract

**Introduction:**

This study investigated factors that influence emergency medicine (EM) patients’ decisions to participate in clinical trials and whether the impact of these factors differs from those of other medical specialties.

**Methods:**

A survey was distributed in EM, family medicine (FM), infectious disease (ID), and obstetrics/gynecology (OB/GYN) outpatient waiting areas. Eligibility criteria included those who were 18 years of age or older, active patients on the day of the survey, and able to complete the survey without assistance. We used the Kruskal-Wallis test and ordinal logistic regression analyses to identify differences in participants’ responses.

**Results:**

A total of 2,893 eligible subjects were approached, and we included 1,841 surveys in the final analysis. Statistically significant differences (p≤0.009) were found for eight of the ten motivating factors between EM and one or more of the other specialties. Regardless of a patient’s gender, race, and education, the relationship with their doctor was more motivating to patients seen in other specialties than to EM patients (FM [odds ratio {OR}:1.752, 95% confidence interval {CI}{1.285–2.389}], ID [OR:3.281, 95% CI{2.293–4.695}], and OB/GYN [OR:2.408, 95% CI{1.741–3.330}]). EM’s rankings of “how well the research was explained” and whether “the knowledge learned would benefit others” as their top two motivating factors were similar across other specialties. All nine barriers showed statistically significant differences (p≤0.008) between EM and one or more other specialties. Participants from all specialties indicated “risk of unknown side effects” as their strongest barrier. Regardless of the patients’ race, “time commitment” was considered to be more of a barrier to other specialties when compared to EM (FM [OR:1.613, 95% CI{1.218–2.136}], ID [OR:1.340, 95% CI{1.006–1.784}], or OB/GYN [OR:1.901, 95% CI{1.431–2.526}]). Among the six resources assessed that help patients decide whether to participate in a clinical trial, only one scored statistically significantly different for EM (p<0.001). EM patients ranked “having all material provided in my own language” as the most helpful resource.

**Conclusion:**

There are significant differences between EM patients and those of other specialties in the factors that influence their participation in clinical trials. Providing material in the patient’s own language, explaining the study well, and elucidating how their participation might benefit others in the future may help to improve enrollment in EM-based clinical trials.

## INTRODUCTION

There is strong evidence that clinical research efforts in emergency medicine (EM) are increasing as demonstrated by the recent establishment of the “Office of Emergency Clinical Research” within the National Institutes of Health. EM research results in special challenges as EM patients present without a pre-existing physician-patient relationship and with acute and often-undifferentiated diseases.^1^,^2^

Many studies have investigated the factors influencing patient participation in clinical trials from different areas of medicine.^3^–^23^ Some of them have attributed decisions regarding participation in clinical research to patients’ gender,^3^–^5^ race,^5^–^8^ linguistic capabilities,^8^–^10^ and socioeconomic status.^11^–^12^ In addition to these personal characteristics, multiple extrinsic factors associated with the research process itself, the clinical staff with whom they interact, the quality of clinical care, and the communication from the research staff are known to play a role.^7^–^10^,^13^–^23^ Some of the reported motivating factors include how well the study was explained to them,^7^,^13^ a strong patient-physician relationship,^7^,^14^–^16^ the knowledge that their participation was going to benefit someone in the future,^7^,^9^,^17^,^18^ and compensation for participating.^19^ Other factors reported to function as barriers to participation include distrust in the doctors,^7^,^20^–^23^ risk of unknown side effects,^7^,^20^–^23^ and language differences.^9^,^10^

Despite this plethora of studies, there are virtually no data investigating whether these factors vary by type of clinical specialty. We hypothesized that, based on specialty, these factors’ influence would indeed vary: e.g., factors influential for oncology patients would not necessarily be similar for obstetric patients. Likewise, EM patients, being under a complex array of physical and psychological stressors, might perceive each motivator and barrier differently when considering participation in a clinical trial.

Since it would be beneficial for EM researchers to know what matters to their patients and how to use this knowledge to customize and optimize their recruitment approach, we sought to determine the factors that influence EM patients’ decision to participate in clinical trials and whether their impact varies from other selected medical specialties.

## METHODS

This was an institutional review board-approved prospective, cross-sectional, self-administered survey study using a convenience sample of patients attending different medical specialties’ outpatient practices at three hospital sites affiliated with a single health network. In preparation, the principal investigator (PI) contacted various specialties for collaboration. Three agreed to participate: obstetrics and gynecology (OB/GYN), family medicine (FM), and infectious disease (ID). The survey subsequently was conducted in the network’s two OB/GYN clinics, four FM clinics, two ID clinics, and three separate emergency departments (EDs). Surveys were offered only to those patients who were in the waiting rooms of these specialties. Thus, patients who came to the ED via ambulance and/or bypassed the waiting room were not surveyed.

Population Health Capsule

What do we already know about this issue?

Enrollment in clinical trials is particularly challenging in EM as patients often present with acute, undifferentiated diseases, and have no preexisting relationship with providers.

What was the research question?

What factors influence EM patients to participate in clinical ttrials, and does their impact vary from other specialties?

What was the major finding of the study?

Impact of several factors that influence EM patients’ participation in research significantly varies from other specialties.

How does this improve population health?

Enrollment in EM research may improve by providing material in patient’s own language, explaining the study well, and elucidating how their participation might benefit others in the future.

The survey was anonymous, voluntary, and administered over a nine-month period (June, 2014 through March, 2015). Potential subjects were approached by multilingual research team members who, in addition to English, were fluent in either Spanish, simplified Chinese, or traditional Chinese. Inclusion criteria required participants to be a minimum of 18 years of age, active patients on the day of the survey, and have the ability to complete the survey without assistance.

The survey was developed by the EM investigators and reviewed by researchers from the other departments involved, a statistician, and the EM research review committee. Based on their feedback, the survey was revised and piloted among 15 randomly selected non-clinical and non-research hospital staff. A brief questionnaire was given to these pilot participants asking whether they could tell us what the purpose of our survey was, how long it took them to complete it, if any questions were too long or confusing, and to provide their general feedback. The respondents accurately determined the purpose of the survey and felt it was appropriate in length. We redistributed the survey to these pilot participants after a two-week interval and compared their second responses to their first to ensure a rate of 95% consistency in interpretation. Revisions were made as needed.

Patients were approached in the “check-in” area of each specialty office by a study team member, were asked their age and, if 18 years of age or older, were offered the survey. Although a cover page explained the purpose of the survey, research staff also provided a brief oral introduction about the survey’s goal, which was to ascertain patients’ opinions about clinical research trials in which doctors test new medications or devices. Confirmation that the patient had not taken the survey on any previous visit to our network’s facilities was obtained. The research staff asked if patients were able to self-administer the survey and, based on their preference, were given a copy of the survey in English or one of the three translated languages. We included in the data analysis only those surveys that indicated the respondent was an active clinic patient.

Regarding potential influential factors for research participation, subjects were asked to rate each factor on a five-point Likert scale as having no (0), very little (1), some (2), moderate (3), or greatest (4) significance. Following the administration of the survey, two trained research associates entered the data into Excel spreadsheets. The PI audited every 20^th^ survey to ensure entry accuracy, consistency of the data entry, and to confirm the integrity of the database.

We compared demographic variables among specialties using a chi-square test. If a significant association (p<0.05) was found, pairwise comparisons were performed to determine which specialties’ results were significantly different from each other. We applied the Bonferroni correction to account for the multiple pairwise comparisons for each demographic variable; with this correction applied, the p-value required for statistical significance was 0.008 (0.05/6).

We used the Kruskal-Wallis test to compare responses for each factor affecting participation in research by specialty. This test is appropriate when comparing two or more groups on an ordinal independent variable.^24^ The Kruskal-Wallis test first ranks the data and then compares the mean of the ranks between groups. If any factor was found to be associated with a specialty, multiple pairwise comparisons were performed to determine which specialties differed. We performed the pairwise comparisons using Dunn’s procedure^25^ with a Bonferroni correction. This method of adjustment is used when dealing with ordinal or non-parametric data with unequal group sizes, if interested in all pairwise comparisons.^26^ The data analysis for this study was generated using SAS version 9.3 (SAS Institute, Cary, NC) and SPSS version 22 (IBM SPSS Statistics for Windows, Armonk, NY, USA).

To further explore the association between the specialty in which the patient took the survey and their responses to specific motivational factors and barriers, we performed an ordinal logistic regression. One motivational factor was chosen, “my relationship with my doctor,” on the premise that responses differed significantly between EM and all other specialties. Similarly, the responses for the two barriers that were chosen, “time commitment” and “religious beliefs,” differed significantly between EM and at least two other specialties. The predictors included in the models were gender, race, education, and specialty. We chose gender, race, and education in an attempt to control for confounding of the relationship between specialty, motivator, barriers to participation in clinical research.

We collapsed the categories for the responses from five to three levels to ensure large enough cell counts for each predictor by each response variable. The collapsed response categories for the motivational factors were “high or very high,” “moderate,” and “slightly or not motivating at all.” Similarly for the barriers, the collapsed categories were “significant or very significant,” “moderate,” and “slight or not a barrier at all.” After collapsing the response variables, we assessed and met the proportional odds assumption.

## RESULTS

We screened 2,917 subjects (Figure), of whom 24 were ineligible due to their age; 2,893 subjects were offered the survey, and 2,025 (70%) agreed to participate. The response rate for EM was 73.3%, for FM 67.2%, for ID 62.8%, and for OB/GYN 76.4% (Table 1). We further excluded 184 surveys because these respondents did not confirm they were active patients. We analyzed the remaining 1,841 surveys.

Demographic characteristics were significantly associated with specialty (p<0.05). Table 2 identifies specific specialties between which an association was observed. Participants from EM (66.4%) and ID (66.3%) were less likely to report better overall health than the participants from FM (71.3%) and OB/GYN (86.1%). Participants from EM and OB/GYN were younger than those in FM or ID. In each specialty, the majority of participants were female (EM, 64.7%; FM, 70.7%; OB/GYN, 99%), except for ID (42.4%). Compared to the other three specialties, participants from EM had a lower education level, a higher percentage reporting Hispanic/Latino ethnicity (EM, 53.2%; FM, 27.8%; ID 29.9%; and OB/GYN, 42.4%), and a higher percentage who said they understood English but had a hard time speaking it (9.1%), along with those who could not speak English (7.3%). While only slightly less than half from EM were White (48.5%), the majority of participants from FM (68.1%), ID (59.2%), and OB/GYN (55.5%) were White.

### Analysis of potential motivating factors

EM patients ranked the majority of motivational factors lower than other specialties. Statistically significant differences were found for eight of the ten motivating factors between EM and one or more specialties. Two factors that did not show statistically significant differences between EM and any other specialty were “my desire to please the doctor” and “the doctor conducting the research is the same race/ethnicity as me.” The top two motivating factors for EM patients appeared to be “how well the research is explained to me” and “knowledge learned from my participation will benefit someone in the future” (Table 3).

### Analysis of potential barriers

All nine barriers showed statistically significant differences between EM and one or more specialties. While all patients, including EM, ranked “risk of unknown side effects” as the strongest barrier, one particular barrier, “my family’s concern,” scored slightly higher for EM patients than other specialties (Table 3).

### Analysis of potential helpful resources

Among six resources assessed that help patients decide whether to participate in a clinical trial, EM patients ranked “having all material provided in my own language” as the most helpful. EM was not statistically different from any other specialty in their rankings of these factors with the exception of whether or not they would be given the opportunity to speak to a patient who has participated in a clinical research study; OB/GYN patients ranked this factor higher (p<0.001) than all other specialties (Table 3).

### Ordinal Logistic Regression Analyses for Selected Factors

Regardless of their gender, race and level of education, patients seen in other specialties had higher odds of being motivated by their relationship with their doctor compared to those seen in EM: FM (OR:1.752, 95% CI[1.285–2.389]), ID (OR:3.281, 95%CI[2.293–4.695]), and OB/GYN (OR:2.408, 95% CI[1.741–3.330]).

Regardless of race, patients seen in FM (OR:1.613, 95% CI[1.218–2.136]), ID (OR:1.340, 95% CI[1.006–1.784]), or OB/GYN (OR:1.901, 95% CI[1.431–2.526]), females (OR:1.322, 95% CI[1.043–1.676]), and those who graduated college or had a higher degree (OR:1.573, 95% CI[1.096–2.256]), had higher odds of stating that time commitment was a barrier than those seen in EM, ID, or men, and those with less than a high school education.

Regardless of the specialty, women (OR:1.505, 95% CI[1.163–1.947]), African Americans (OR:1.903, 95% CI[1.400–2.587]), Hispanics (OR:1.724, 95% CI[1.306–2.276]), multiracial patients (OR:1.761, 95% CI[1.060–2.926]), and patients of other races (OR:2.362, 95% CI[1.547–3.607]) all had higher odds of stating that their religious beliefs were more of a barrier when compared to male Whites. Patients who were college graduates or had a higher degree (OR:0.569, 95% CI[0.393–0.823]), as well as those with some college or a two year degree (OR:0.644, 95% CI[0.463–0.897]), had lower odds of their religious beliefs being a barrier compared to those with less than a high school diploma.

## DISCUSSION

Although EM treats a large and diverse population, including women, pediatric, geriatric, and patients of color, and has the potential to promote diversity in clinical trials, recruiting patients for participation in EM clinical trials appears to be very challenging.^1^,^2^ To identify factors that influence EM patients’ decision to participate in clinical trials and to assess whether the impact of these factors varies from other specialties, we conducted this study among patients visiting EDs and compared their responses with the responses of patients attending a broad range of other medical specialties. For example, FM provided patients to our sample that – in contrast to EM – present for primary care and typically have established relationships with their providers. Further, inclusion of patients from ID (the AIDS Activity Office/Hepatitis Care Center and the Travel ID Clinic) added those who were suffering from contagious illnesses, and patients from OB/GYN represented a vulnerable population in our sample. Despite the heterogeneity of the populations compared from these four specialties, acceptance rates for all specialties were satisfactory except for ID. Since the reason(s) for non-participation were not collected and also because IRB restrictions do not permit collecting demographics on non-participants, a non-response bias analysis was not possible.

Compared to OB/GYN and ID patients, EM and FM patients did not indicate strong motivation to participate in clinical trials by the factors listed in our survey. It is noteworthy that since the survey was offered to all patients in the waiting rooms of each specialty, the survey was consequently not offered to those EM patients who arrived by ambulance. Other patients who were not surveyed might have included those whose condition was so severe that they were taken directly to a bed without any wait. However, had we included these patients, it is possible that the motivational factor scores for EM may have been even lower because of the physical and psychological stressors associated with these patients’ severe health conditions.

Based on median scores, ID and OB/GYN patients ranked a total of four out of the 10 motivational factors as very highly motivating, while FM patients ranked three, and EM patients ranked only two factors as very highly motivating. For EM patients, scores for motivating factors were lower, and scores for most barriers were equal to other specialties. These findings help explain the challenges EM investigators and research staff experience and struggle with when trying to enroll patients in EM-based clinical trials.

It perhaps is not surprising that differences exist among the various specialties in their perceptions of factors that both motivate and deter patients from participating in clinical trials. In fact, EM patients are less likely to be influenced by the doctor’s reputation or their relationship with their physician. This is in contrast to prior studies that have generally described an established relationship with the investigating physician as a strong motivator in making medical decisions, including clinical research participation.^14^–^16^ EM patients being less influenced by these two factors may be due to the fact that they neither have an established relationship with their emergency care provider nor time to check on the provider’s reviews.

The motivation for all patients, including EM, appeared to be the least affected by the investigator’s race, their desire to please the doctor, financial compensation, and the investigator’s gender (with the exception of OB/GYN patients). The factor “knowledge gained will benefit someone in the future” was ranked highly by all specialties. Altruism is widely reported to be a motivating factor for research participation,^9^,^17^ and this finding supports a recent study conducted by Limkakeng, et al. that identified altruism as a motivating factor for research participation by the EM population.^18^

The primary barrier to participation for all specialties, including EM, is “fear of unknown side effects.” This finding is in concordance with other reports.^20^–^23^ It is unclear to what extent this barrier could be mitigated by emphasizing better and/or more complete communication with potential enrollees. “My family’s concerns” was the second strongest barrier for EM patients. Involving families in decision-making in EDs has been reported to be challenging.^27^ It is possible that family members are not present with the patient, or if they are present, they may be under as much psychological stress and anxiety as the patients themselves.

Factors that are easily modifiable by investigators, such as the provision of written or electronic material, were rated as moderately helpful in recruiting for clinical research. This was true even for the provision of material in the patient’s own language. Compared to other specialties, EM had more respondents who belonged to a Latino or Hispanic origin, had less than a college-level education, and had fewer participants who were fluent in English. A prior study regarding enrollment challenges in EM research reported that a sizable proportion of eligible, non-English-speaking Latinos were not enrolled due to language barriers.^1^ The availability of translated material has been noted as an effective measure in overcoming linguistic barriers.^8^–^10^ In fact, the availability of translated surveys allowed a high percentage of EM respondents (20.1%) to participate in the current study. Further, the number of translated surveys used for each specialty corresponded with the number of those who were not proficient in the English language.

As the time frame to consent patients in the ED is usually shorter than for other specialties,^27^ and the time taken for an explanation of research could delay the immediate clinical intervention,^28^ the availability of translated material could help to improve EM study enrollment. Other potential solutions that may address the challenge of enrolling EM patients include conducting less complex, shorter intervention studies,^2^ and either waiving or allowing deferred consent in EM clinical trials.^29^, ^30^

Since demographic variables, such as patient’s gender,^3^–^5^ race,^5^–^8^ linguistic capabilities,^8^–^10^ and socioeconomic status^11^,^12^ are known to influence their decisions to participate, the heterogeneity of the study sample should not be undermined when interpreting these results. However, it is noteworthy that even though EM had a large female population, the results for EM were still significantly different from OB/GYN for six out of 10 motivating factors and for six out of nine barriers. While OB/GYN patients ranked a majority of motivating factors higher than the other specialties, their scores for barriers were higher as well. This, to some extent, explains the underlying reasons of gender disparity in clinical trials. ID (which had more male respondents than other specialties) appeared to be the second most motivated, after OB/GYN patients, to participate in research, but they were less deterred by barriers when compared to all other specialties. This may indicate that individuals with serious infections have better chances to participate, given the importance of research trials in these areas.

Our hypothesis that the impact of factors influencing a patient’s decision to participate in clinical trials may vary among specialties was tested and confirmed by logistic regression analyses of a few of those factors that were significantly different for EM from either all, or a majority of other specialties in our sample. Regardless of their gender, race, and education status, “relationship with their doctor” was least motivating to EM patients than those from FM, ID, and OB/GYN. This finding as discussed above is logical. Patients and emergency physicians usually see each other only one time, and the chance of them seeing each other again in the future is slim to none. Additionally, EM patients have no choice of selecting their own doctor, whereas in other specialties, patients routinely make appointments with their preferred doctor for their follow-up care.

The results were similar for the barrier of “time commitment.” Regardless of patient’s race, “time commitment” was considered less of a barrier to EM patients, males, and those with less than a high school diploma. As the majority of EM patients present with acute and often-undifferentiated illnesses, it is logical that for them the diagnosis and resolution of emergency take priority over time commitment. Regression analysis of a second barrier, “my religious beliefs,” which was scored significantly differently by EM patients than two other specialties (FM and ID), was found to be influenced by factors other than the specialty.

The results confirm our hypothesis and show that, regardless of demographic characteristics, the impact of some influential factors does vary from one specialty to the other. Therefore, we recommend that researchers customize their recruitment approach according to their specialty.

### Strengths and Limitations

To our knowledge, this is the first large-scale prospective study that investigated factors that influence EM patients’ decision to participate in clinical trials. This is also the first one indicating that the impact of the same factor may vary from one specialty to another. The strengths of this study include its large number of patients and an excellent response rate. Also, the availability of multilingual research staff and translated surveys in Spanish, simplified Chinese, and traditional Chinese maximized diversity.

Although surveying previous clinical trial participants and those who declined to participate in previous clinical trials may have been a better option for investigating influential motivators and barriers, human research participant protection and confidentiality-related policies did not allow us to identify and survey this population. The current study used a convenience sampling method based on when the research staff was available and on the patients who happened to visit the office that day, rather than a random-selection method. In addition, all participants were approached in the waiting room of these specialties. Since ambulance patients were excluded, our findings do not represent this subgroup of ED patients.

We acknowledge that although the overall response rate was satisfactory, a non-response bias may have potentially swayed the results. However, the reason(s) for non-participation were not collected, and IRB restrictions did not permit collecting demographics on non-participants. Therefore, a non-response bias analysis was not possible. Further, the decision to participate in a clinical trial depends on a variety of factors, and it is possible that a clinical trial has other motivators and barriers that were not assessed in this study. We acknowledge our survey responses may not mimic actual responses of potential subjects to a legitimate research trial invitation.

## CONCLUSION

Even though a patient’s decision to participate in clinical trials depends on multiple factors, we conclude that the impact of the same factor may vary from one specialty to another. Researchers should focus on factors that are more influential to their specialty populations and should customize study designs to make clinical trials more appealing to potential participants. When considering participation in clinical research, EM patients ranked their relationship with the doctor and the importance of their physician’s reputation as significantly less important than patients in other specialties. The fear of unknown side effects was the most significant barrier for patients of all specialties. Although compared to other selected specialties, EM patients appeared to be less motivated on most factors assessed, providing material in a patient’s own language, explaining the study well, and elucidating how their participation might benefit others in the future, may improve enrollment in EM-based clinical trials.

## Figures and Tables

**Figure:**
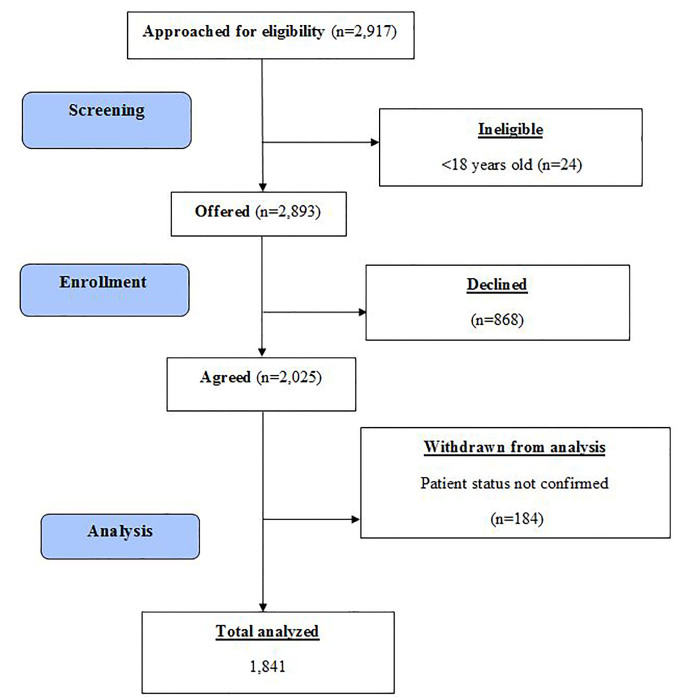
CONSORT flow diagram.

**Table 1:** Response rate of emergency medicine (EM), family medicine (FM), infectious disease (ID), and obstetrics/gynecology (OB/GYN) patients to a survey regarding participation in clinical trials.

	EM	FM	ID	OB/GYN
Offered	726	734	693	740
Agreed	532	493	435	565
Response rate %	73.3	67.2	62.8	76.4

**Table 2:** Demographics of emergency medicine (EM), family medicine (FM), infectious disease (ID) and obstetrics/gynecology (OB/GYN) respondents.

Variables (total respondents)	EM n(%) 457 (24.8)	FM n (%) 451 (24.5)	ID n (%) 408 (22.2)	OB/GYN n (%) 525 (28.5)	*p* value‡
Self-reported health status (1,835)					<0.001
Poor/fair	153 (33.6)^a^*	129 (28.7)^a^	137 (33.7)^a^	73 (13.9)^b^	
Good/very good/excellent	302 (66.4)	320 (71.3)	269 (66.3)	452 (86.1)	
Age (1,774)					<0.001
Under 35	207 (46.6)^a^	96 (22.2)^b^	60 (15.2)^c^	381 (75.8)^d^	
35–65	206 (46.4)	268 (62.0)	285 (72.2)	120 (23.9)	
>65	31 (7.0)	68 (15.7)	50 (12.7)	2 (0.40)	
Gender (1,779)					<0.001
Male	158 (35.4)^a^	127 (29.3)^a^	228 (57.6)^b^	5 (1.0)^c^	
Female	289 (64.7)	307 (70.7)	168 (42.4)	497 (99.0)	
Highest education level (1,756)					<0.001
Less than high school diploma	86 (19.7)^a^	40 (9.4)^b^	80 (20.5)^c^	66 (13.2)^b,c^	
High school graduate or GED	187 (42.8)	137 (32.0)	121(31.0)	164 (32.8)	
Some college or 2-year degree	113 (25.9)	129 (30.1)	116 (29.7)	170 (34.0)	
College graduate or more	51 (11.7)	122 (28.5)	74 (18.9)	100 (20.0)	
Latino or Hispanic origin? (1,757)					<0.001
Yes	235 (53.2)^a^	119 (27.8)^b^	117 (29.9)^b^	210 (42.4)^c^	
No	207 (46.8)	309 (72.2)	275 (70.2)	285 (57.6)	
Race (1,643)					<0.001
White or Caucasian	197 (48.5)^a^	280 (68.1)^b^	218 (59.2)^c^	254 (55.5)^a,c^	
Black or African-American	52 (12.8)	37 (9.0)	66 (17.9)	62 (13.5)	
Hispanic, Puerto Rican, Latino, Columbian, Spanish, Mexican-American, Dominican	114 (28.1)	59 (14.4)	56 (15.2)	88 (19.2)	
Multi-racial	19 (4.7)	13 (3.2)	7 (1.9)	23 (5.0)	
Other than above	24 (5.9)	22 (5.4)	21 (5.7)	31 (6.8)	
Speak and understand english? (1,753)					<0.001
Very well	335 (75.7)^a^	361 (83.4)^b,c^	305 (78.8)^a,c^	432 (87.6)^b^	
Pretty good	35 (8.0)	38 (8.8)	35 (9.0)	25 (5.1)	
Can understand, but have a hard time speaking it	40 (9.1)	26 (6.0)	26 (6.7)	20 (4.1)	
Cannot speak English	32 (7.3)	8 (1.9)	21 (5.4)	16 (3.3)	
Employment (1,735)					<0.001
Full-time	147 (34.0)^a^	174 (40.6)^b^	113 (29.4)^c^	187 (38.3)^d^	
Part-time	61 (14.1)	55 (12.8)	41 (10.7)	100 (20.5)	
Unemployed and looking for work	80 (18.5)	37 (8.6)	41 (10.7)	72 (14.8)	
Unemployed, but not looking for work	36 (8.3)	28 (6.5)	39 (10.1)	67 (13.7)	
Student	12 (2.8)	11 (2.6)	8 (2.1)	20 (4.1)	
Retired	48 (11.1)	82 (19.1)	73 (19.0)	8 (1.6)	
Other	18 (4.1)	8 (1.9)	19 (4.9)	21 (4.3)	
Disabled	31 (7.2)	34 (7.9)	51 (13.7)	13 (2.7)	
Income in 2013 (1,680)					<0.001
Less than $30,000	237 (56.4)^a^	186 (44.5)^b^	191 (51.9)^b, c^	250 (52.7)^a, c^	
$30,001–$50,000	52 (12.4)	78 (18.7)	46 (12.5)	80 (16.9)	
$50,001–$75,000	14 (3.3)	34 (8.1)	28 (7.6)	33 (7.0)	
More than $75,001	12 (2.9)	48 (11.5)	32 (8.7)	18 (3.8)	
I‘d rather not answer	105 (25.0)	72 (17.2)	71 (19.3)	93 (19.6)	
Respondents using translated surveys	92 (20.1)	33 (7.3)	51 (12.5)	30 (5.7)	

*GED*, General Education Development.

* Superscript letters highlight results of pairwise comparisons by specialty. For each demographic variable, cells with at least one letter the same indicate specialties for which there was no statistically significant association.

‡ P-values are the results of the omnibus chi-square test for each demographic variable by specialty and are not adjusted in any way.

**Table 3:** Mean response to each motivational factor, barrier, and helpful resource for emergency medicine (EM), family medicine (FM), infectious disease (ID), and obstetrics and gynecology (OB/GYN) respondents to a survey regarding participation in clinical trials.

Variables	EM	FM	ID	OB/GYN
Motivational factors				
My relationship with my doctor	2.54*^a^	2.99^b^	3.35^c^	3.16^b,c^
Doctor’s reputation in the community	2.6^a^	3.01^b^	3.11^b^	3.37^c^
How well the research is explained to me	3.02^a,c^	3.21^c^	3.35^b,c^	3.44^b^
My desire to please the doctor	1.59^a,b^	1.36^a^	1.73^b^	1.57^a,b^
Money offered for my participation	1.84^a^	1.77^a^	1.89^a,b^	2.17^b^
A friend or family member participating in the same study	1.73^a,b^	1.6^b,c^	1.39^c^	1.89^a^
The doctor conducting the research is the same gender (sex) as me	1.15^a^	1.07^a^	1.02^a^	1.56^b^
The doctor conducting the research is the same race/ethnicity as me	0.86^a^	0.71^a^	0.73^a^	0.96^a^
The doctor conducting the research speaks the same language as I do	1.79^a^	1.81^a^	2.04^a,b^	2.23^b^
Knowledge learned from my participation will benefit someone in the future	2.94^a^	3.05^a^	3.32^b^	3.18^a,b^
Barriers				
My distrust in doctors	1.85^a^	1.79^a^	1.66^a^	2.36^b^
Time commitment	2.17^a^	2.56^b^	2.28^a^	2.74^b^
My family’s concern	2.4^a^	2.26^a,b^	1.99^b^	2.32^a^
My religious beliefs	1.56^a^	1.21^b^	1.23^b^	1.58^a^
Clinical research studies are too hard to understand	1.42^a^	1.28^a^	1.26^a^	1.69^b^
Study related phone calls for follow-ups	1.7^a^	1.65^a^	1.53^a^	1.99^b^
Multiple follow-up visits related to the study	1.88^a^	2.11^a,b^	1.84^a^	2.35^b^
Risk of unknown side effects	2.78^a,c^	3.07^c,b^	2.59^a^	3.25^b^
Access to transportation	1.78^a^	1.46^b^	1.64^a,b^	1.8^a^
Helpful resources				
Written material explaining the research study	2.8^a^	3.0^a^	3.0^a^	3.02^a^
DVDs or electronic material explaining the research study	2.6^a^	2.74^a^	2.7^a^	2.82^a^
Having opportunity to speak to a patient who has participated in a clinical research study	2.66^a^	2.73^a^	2.63^a^	3.04^b^
Having access to a support group of patients who have participated in clinical research	2.52^a^	2.51^a^	2.44^a^	2.67^a^
Having all material provided in my own language	3.01^a^	3.0^a^	3.09^a^	3.24^a^
Having access to a medical interpreter throughout the study	2.38^a^	2.3^a^	2.23^a^	2.51^a^

* Values with at least one letter the same indicate specialties for which there was no statistically significant association as determined by pairwise comparisons of mean ranks.
